# Plants, Birds and Butterflies: Short-Term Responses of Species Communities to Climate Warming Vary by Taxon and with Altitude

**DOI:** 10.1371/journal.pone.0082490

**Published:** 2014-01-08

**Authors:** Tobias Roth, Matthias Plattner, Valentin Amrhein

**Affiliations:** 1 Hintermann & Weber AG, Reinach, Switzerland; 2 University of Basel, Zoological Institute, Basel, Switzerland; 3 Research Station Petite Camargue Alsacienne, Saint-Louis, France; Institute of Botany, Czech Academy of Sciences, Czech Republic

## Abstract

As a consequence of climate warming, species usually shift their distribution towards higher latitudes or altitudes. Yet, it is unclear how different taxonomic groups may respond to climate warming over larger altitudinal ranges. Here, we used data from the national biodiversity monitoring program of Switzerland, collected over an altitudinal range of 2500 m. Within the short period of eight years (2003–2010), we found significant shifts in communities of vascular plants, butterflies and birds. At low altitudes, communities of all species groups changed towards warm-dwelling species, corresponding to an average uphill shift of 8 m, 38 m and 42 m in plant, butterfly and bird communities, respectively. However, rates of community changes decreased with altitude in plants and butterflies, while bird communities changed towards warm-dwelling species at all altitudes. We found no decrease in community variation with respect to temperature niches of species, suggesting that climate warming has not led to more homogenous communities. The different community changes depending on altitude could not be explained by different changes of air temperatures, since during the 16 years between 1995 and 2010, summer temperatures in Switzerland rose by about 0.07°C per year at all altitudes. We discuss that land-use changes or increased disturbances may have prevented alpine plant and butterfly communities from changing towards warm-dwelling species. However, the findings are also consistent with the hypothesis that unlike birds, many alpine plant species in a warming climate could find suitable habitats within just a few metres, due to the highly varied surface of alpine landscapes. Our results may thus support the idea that for plants and butterflies and on a short temporal scale, alpine landscapes are safer places than lowlands in a warming world.

## Introduction

Among the currently occurring changes in environmental conditions, climate warming presumably has the greatest potential to change species communities [1], [2]. An apparent response to climate warming is that species shift their distribution towards higher latitudes or altitudes [3]–[6]. However, species seem to vary greatly in their range shifts, probably depending on the particular traits of the species [7]. For instance, the differential mobility of taxa such as plants or birds likely predicts the rate at which they can track climate change [8], [9].

Yet, whether the response to climate change of different taxonomic groups is constant over larger environmental ranges is currently unclear [8]. Our lack of knowledge is particularly evident with regard to responses to climate warming across altitudinal ranges [10]. It has been suggested that lowland forests are one of the least reactive terrestrial ecosystems and are particularly threatened by climate warming, because adaptation of communities lags behind environmental change [11]. Other studies proposed that mountain ecosystems are particularly threatened [10], [12], e.g. because climate warming causes a significant upward shift in optimum habitat of species, leading to decreasing species ranges, because land area is usually decreasing with altitude [13], [14]. Recently, however, it was suggested that the velocity of temperature change is lowest in alpine landscapes [15]. Further, alpine landscapes could be relatively safe places in a warming world, because in the highly varied surface of alpine landscapes, thermal mosaics usually create fine-scale habitats inhabited by species with different thermal preferences; thus, in a warming climate, many alpine plant species could find suitable habitats fitting their thermal preferences within just a few metres [16].

Here, we used data from the Swiss biodiversity monitoring program [17], [18] that were collected over the diverse altitudinal gradients but small latitudinal ranges of Switzerland. We studied temporal changes in communities of vascular plants, butterflies and breeding birds over an altitudinal range of about 2500 metres. Data were collected in 214 1-km^2^ sample squares that were regularly distributed over the entire country. Sample squares were surveyed twice between 2003 and 2010, with five years between two surveys of a sample square. For all three species groups, data were collected on the same study sites during the same years, and thus, communities of the three species groups largely experienced the same changes in environmental conditions.

To measure whether communities changed towards warm-dwelling species, we used the recently developed community temperature index CTI [9]. For this index, each species is given an indicator value reflecting its temperature niche on a national or continental scale; the CTI then describes a community as the average of the individual indicator values of the recorded species [9], [19]. A low CTI would thus reflect a large proportion of low-temperature dwelling species, and a temporal increase of CTI would indicate that the proportion of high-temperature species has increased. Unlike traditional measures such as species richness, the CTI accounts for species-specific sensitivity to temperature: if in a community a warm-dwelling species were replacing a cold-dwelling species, the CTI would increase, while a traditional measure such as species richness would remain constant. Furthermore, we extended the current CTI framework by additionally inferring the variation of temperature indicator values of the individual species present in a community, which we call the community temperature variation CTV. Using the CTV, we aimed to test whether as a response to climate warming, communities tended to become more homogeneous with respect to temperature niches of species [20].

Following the argument by Scherrer & Körner [16] that in the varied alpine terrain, many plant species could find habitats with suitable micro-climatic conditions within just a few metres, we predicted that CTIs of plants would change at a slower rate in alpine environments than in lowlands. However, different species groups are likely to respond to environmental factors at different spatial scales, with important consequences on how they may react to climate change [2], [21]. For example, birds and butterflies are among the most dispersive species, so they should be able to track climate change more easily than plants [8]. Further, given that birds are to a large extent airborne organisms and thus are probably influenced more by air temperatures than by micro-climatic conditions, we predicted that community changes in alpine environments are larger in birds than in plants. Predictions for butterflies are less straightforward, because while being generally mobile, butterflies strongly depend on their relatively sedentary host plants both for feeding and reproduction [22]. We therefore expected butterflies to show a response to climate change that is intermediate between plants and birds.

## Materials and Methods

The study took place between 2003 and 2010 in Switzerland. About 70% of Switzerland is mountainous, with the Alps covering about 60% and the Jura Mountains covering about 10% of the country. Overall, Switzerland covers altitudes from 193 to 4634 m. In Switzerland, temperatures increased from 1959 to 2008 at all altitudes, with an average warming rate of 0.35°C per decade, which is about 1.6 times the northern hemispheric warming rate [23].

### Ethics statement

No specific permits were required for the described field studies, as plants, birds and butterflies were surveyed along existing trails that are not privately owned. The field studies did not involve collecting of endangered or protected species, except for rare cases in butterflies where a few specimens of faunistic interest were collected with the permission of the Swiss Federal Office for the Environment (FOEN).

#### Swiss biodiversity monitoring scheme

**Figure 1:**
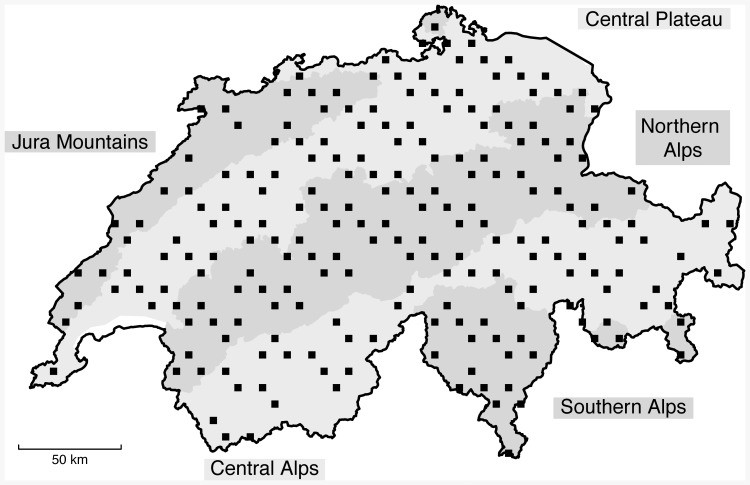
Distribution of sample sites over Switzerland. Locations of the 214 analysed 1-km^2^ sample squares from the Swiss national biodiversity monitoring program for which data for all three species groups were available (vascular plants, butterflies and breeding birds).

We used data from the Swiss Biodiversity Monitoring scheme (BDM, www.biodiversitymonitoring.ch) that was launched in 2001 to monitor Switzerland's biodiversity and to meet the Convention on Biological Diversity of Rio de Janeiro [18]. Fieldwork was done using standardised protocols (Text S1). For the BDM scheme, 428 sample squares of 1 km^2^ were selected that were regularly distributed and aligned within the approximately 41'295 km^2^ units of the Swiss national coordinate system. Excluding sample squares of 100% water surface, as well as sample squares that were too dangerous to do field work because of their exposed alpine terrain, 396 squares were surveyed for the presence of vascular plants, butterflies and breeding birds. Each year, one fifth of sample squares were surveyed, chosen to constitute a regularly spaced subsample of all sites, and each site was surveyed every five years. Between 2003 and 2010, three fifths of sample squares were thus surveyed twice. From the 237 sample squares on which two surveys were done, we excluded *a priori* 23 squares because surveys did not meet our standards of data collection or of weather conditions according to the protocol (Text S1). We analysed data from 214 sample squares (Fig. 1). Average altitudes within the 214 sample squares ranged from 263 m to 2840 m, and mean ± SD altitude of sample squares was 1190±693 m. In Switzerland, the tree line varies in altitude from 1750 m above sea level in the northern front ranges to 2350 m in some parts of the central Alps [24]. Of the 214 sample squares, 22 (10%) were mostly above 2350 m. Average (± SD) numbers of species counted at a sample square during the first survey were 220.4±59.5 for vascular plants, 28.6±15.1 for butterflies, and 33.2±12.6 for birds, and during the second survey 228.1±59.9 for vascular plants, 28.5±14.1 for butterflies, and 32.6±12.5 for birds.

#### Temperature data

To examine possible altitudinal effects on the changes of air temperatures over the years, we used data on air temperatures from the 14 meteorological stations that were freely available from the Federal Office of Meteorology and Climatology [25]. These 14 meteorological stations were selected by the Federal Office of Meteorology and Climatology to represent the different climatic regions of Switzerland [26], and stations were distributed over an altitudinal range from 273 to 2501 m, with an average (± SD) of 1042±767 m. We present results on air temperatures to aid the interpretation of the results on temporal change of species communities, but note that air temperature data were not directly used in the analyses on species communities (see below).

Because survey methods for temperature differed among meteorological stations (e.g., regarding the number of measurements taken per day), the time-series of temperatures were homogenized using the method for homogenization of monthly data series as described in Begert et al. [25]. As community changes usually lag behind climate change [8], [9], we decided haphazardly to examine temperature data for a period from 1995 to 2010 that was twice as long as our study period on community change that lasted from 2003 to 2010. Further, since different species may react to different aspects of temperature, we examined for a given year both the mean of the monthly temperature averages from April to September, which is the period when data on species communities were collected, and the mean temperature of the coldest month. We chose these two measures of temperature because we believe that they are likely to be relevant for many species under study, but we acknowledge that they may not be appropriate for all species. To analyse the two measures of temperature, we used separate linear mixed models (LMMs) with either the average temperature from April to September or the average temperature of the coldest month as dependent variable and altitude and linear trend as well as their interaction as predictor variables. Because temperature measures taken from the same meteorological stations and measures taken in the same years are statistically dependent, we used meteorological station and year as random factors in the LMMs. We then tested whether the temporal trends of temperature measures differed among altitudes (interaction temporal trend×altitude) and whether mean air temperatures were increasing over years (main effect temporal trend).

#### Species temperature index STI

Analyses on species communities were based on a recently developed framework to measure change in community composition in response to climate warming [9]. The framework is centred on species-specific long-term average temperatures that are experienced by individuals of a species over its larger (e.g., national or continental) range, which is the species temperature index (STI). The STI is a species-specific measure of the temperature niche of a species [9]. For the species investigated in this study, we used STI values that were successfully applied in other studies [8], [9], [16]. For plant STIs, we used Ellenberg species indicator values for temperature that were developed for Switzerland [27]. Ellenberg temperature values are based on expert knowledge (values 1–5 in 0.5 steps), and recent studies showed that they give reasonable results on conditions at patches of habitat even at a very fine spatial scale [16]. For butterflies and birds, we used STIs obtained as the mean temperature at which a butterfly or breeding bird species occurred in Europe (for our sources of butterfly and bird STI values, see [28] and Acknowledgements). We used Settele et al. [29] as reference for the distribution of butterflies, and Hagenmeijer & Blair [30] for birds. Three butterfly species were excluded *a priori* from the analyses because they are largely wandering species in Switzerland (*Colias crocea*, *Vanessa atalanta* and *Vanessa cardui*).

#### Community temperature index CTI and community temperature variation CTV

Any local species assemblage can be characterized by a community temperature index (CTI) calculated as the average of the species temperature indices (STI) of the species recorded in the assemblage [9]. A low CTI would thus reflect a large proportion of low-temperature dwelling species (i.e. species with low temperature indices STI), and an increase of CTI over time would indicate that the proportion of species with higher temperature niches has increased. In site-based approaches such as the CTI, mean values of all species per site are often calculated taking into account the abundances of the species [9], [31]; however, this leads to abundant species having larger influence on the results than rare species [31]. Because we aimed at measuring a community response to climate change that is similarly influenced by common and by rare species, we based our calculations of CTIs on occurrence (presence/absence) data and did not weight them with the abundance of a species. Note, however, that when accounting for the abundance of a species, presence-absence based CTIs are usually very similar to the results obtained from CTIs based on occurrence data [8], [9].

We extended the current CTI framework to test whether as a response to climate warming, communities tended to become more homogeneous with respect to temperature niches of species. We used the standard deviation of species temperature indices (STI) of the species recorded in a community at a sample square as our measure of community temperature variation (CTV). CTV values are large if the range of temperature niches of species occurring in a community is broad. Community averages as given by the CTI and community variation as given by the CTV are complementary measures and may reveal different patterns: For instance, if in a community, there were warm-dwelling species invading, the CTV would increase, and if there were cold-dwelling species disappearing, the CTV would decrease; in both cases, the CTI would increase. Note, however, that particularly in plants where species temperature values were restricted to discrete values between one and five (see above), CTI and CTV may be inherently correlated to some extend because communities with CTIs close to one or five can vary less than communities with intermediate CTI values.

#### Statistical analysis

For each sample square *i* and each species group, we calculated
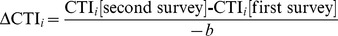


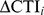
 is thus a measure of the temporal change of local species composition from the first to the second survey. If 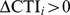, then the species community at a sample square *i* changed towards warm-dwelling species from the first to the second survey, and if 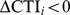, then the species community at a sample square *i* changed towards cold-dwelling species. Because we aimed at comparing changes in species composition between species groups, and because the methods for obtaining species temperature indices (STI) differed among species groups (see above), we standardized the change in species composition using the group-specific constant *b*. *b* is the slope of the group-specific linear regression of CTI values from the first survey on altitude and was −9.2×10^−4^, −1.2×10^−3^ and −5.4×10^−4^ for plants, butterflies and birds, respectively.

Using standardized 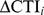 values, local changes in CTI can be interpreted as the difference in altitude in metres needed to go uphill or downhill to find the same difference in CTI as we measured for the temporal change in CTI at a sample square. For example, a constant *b* of −0.001 for butterflies means that the CTI of butterflies on average decreases by 0.001 per metre increase in altitude. If at a sample square, we would find a temporal change in CTI of 0.05, then, on a national or continental scale, we would on average need to go 0.05/−0.001 = −50 m downhill to find the same CTI with more warm-dwelling species as we found at our sample square at the second survey as compared to the first survey. This would mean that at our sample square, the butterfly community showed an uphill shift of 50 m between 2003 and 2010 (or more exactly, in the five years from the first to the second survey at the particular sample square).

For the community temperature variation CTV, we calculated for each sample square *i* and each species group
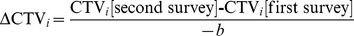
Here, the constant *b* is the slope of the group-specific linear regression of CTV values from the first survey on altitude and was −8.6×10^−5^, −5.4×10^−4^ and −3.9×10^−4^ for plants, butterflies and birds, respectively. If 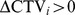, then the temperature niches of the species present in the community at a sample square *i* became more variable from the first to the second survey, and if 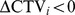, then the temperature niches of the species present in the community at a sample square *i* became more homogenous.

To test whether standardized local changes in community average (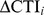) or in community variation (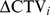) depended on altitude, we used linear models with 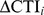 or 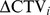 as dependent variables and with linear, quadratic and cubic polynomials of altitude as independent variables. To control for a possible confounding effect of altitudinal range within a sample square, we added altitudinal range (m) within a sample square as a covariate. For the LMMs, we subtracted 500 m from the true altitude of each sample square, which shifts the intercept of the model from 0 m to 500 m. Consequently, the estimated value for the intercept obtained from the LMMs reflected CTI and CTV predictions for a community at an altitude of 500 m, which is about the average altitude of the central plateau in Switzerland (Fig. 1). To predict CTIs and CTVs for communities at the upper limit of the tree line in Switzerland (about 2350 m in the central Alps [24]), we made model predictions for an altitude of 2350 m.

It seems likely that the CTI or CTV in an assemblage of many species is more precise than the measure of CTI in an assemblage of fewer species. We therefore expected that the residual variation in our linear models would decrease with increasing species richness. As this would violate the assumption of homogeneity of variances [32], we used the gls-function of the R-package nlme [33] and followed the protocol as proposed by Zuur et al. [32] to account for heterogeneity of residuals: first, we used full models that included linear, quadratic and cubic polynomials of altitude as well as the altitudinal range within squares and tested three different variance-covariance structures, i.e. fixed variance (like in traditional linear models), power of species richness, and constant plus power of species richness [32]. We then selected the variance structure of the model with the lowest AIC value and visually checked the residuals for heterogeneity and other model violations. Second, to select the model on which we based inference, we started with the full model that included linear, quadratic and cubic polynomials of altitude as well as the altitudinal range and the respective variance-covariance structure found during the first step. We backward selected based on AIC values to obtain the minimal adequate model. Third, likelihood ratio tests using restricted maximum likelihood were performed to test for significance of the independent variables; restricted maximum likelihood is used in mixed models to correct the estimator for the variance [32]. Finally, to obtain p-values and confidence intervals for model predictions, we used bootstrap methods with 1000 iterations [32].

To analyse the temporal trends of air temperatures, we used the lmer-function of the R-package nlme [33]. All analyses were performed using the software R [34].

## Results

Our results are based on the assumption that community temperature index (CTI) and community temperature variation (CTV) are accurate descriptions of the average and variation of temperature niches of species in the local communities. If this assumption is correct, then CTI and CTV values of different species groups in local communities that experienced the same climatic conditions should be positively correlated. In our case, the three species groups were surveyed on the same study sites during the same years, and indeed, community averages (CTIs) of species groups at the 214 sample squares were strongly positively correlated (Pearson's correlation of CTIs of first surveys of each sample square; plants-butterflies: r = 0.97, t = 54.8, d.f. = 212, p = <0.001; plants-birds: r = 0.83, t = 21.7, d.f. = 212, p = <0.001; butterflies-birds: r = 0.81, t = 20.4, d.f. = 212, p = <0.001). Likewise, the community variations (CTVs) of the species groups were positively correlated (plants-butterflies: r = 0.69, t = 13.7, d.f. = 212, p = <0.001; plants-birds: r = 0.38, t = 5.6, d.f. = 212, p = <0.001; butterflies-birds: r = 0.67, t = 13.0, d.f. = 212, p = <0.001).

The temporal changes of community average (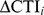), however, differed between species groups and were thus not significantly correlated (plants-butterflies: r = −0.06, t = 0.9, d.f. = 212, p = 0.37; plants-birds: r = −0.05, t = 0.7, d.f. = 212, p = 0.50) or were even negatively correlated (butterflies-birds: r = −0.22, t = 3.3, d.f. = 212, p = 0.001). Similarly, the temporal changes of community variation (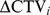) were not significantly correlated between species groups (plants-butterflies: r = 0.02, t = 0.4, d.f. = 212, p = 0.72; plants-birds: r = 0.02, t = 0.2, d.f. = 212, p = 0.81; butterflies-birds: r = −0.01, t = 0.1, d.f. = 212, p = 0.94).

**Figure 2:**
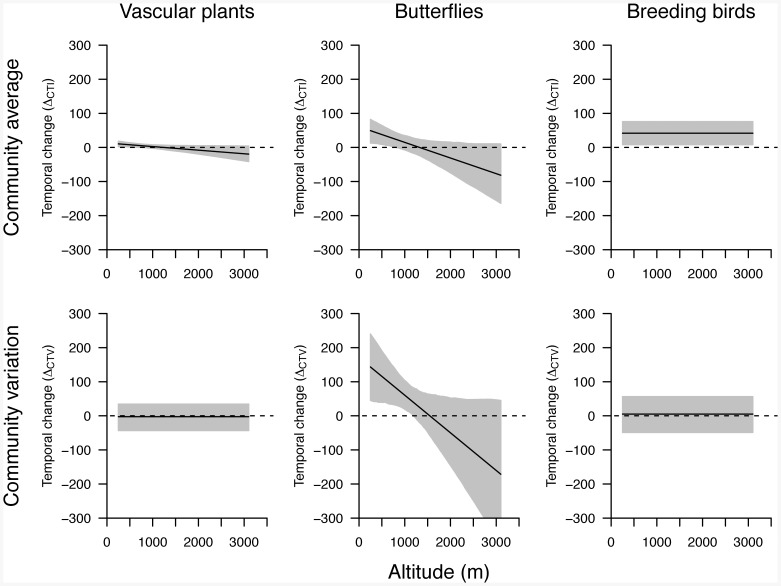
Temporal change of temperature indices of plant, butterfly and bird communities. Given are model predictions for temporal changes of community average of temperature indices (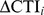, upper panels) and of community variation in temperature indices (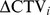, lower panels) between two surveys at a sample square *i* separated by five years within the period 2003–2010, across the altitudinal range covered in the Swiss national biodiversity monitoring program. Black lines are regression lines from minimal adequate linear models, and grey areas represent bootstrapped 95% confidence intervals. Predicted values with confidence intervals that do not include zero are judged as being significantly different from zero.

We found that at low altitudes, CTIs of vascular plants, butterflies and birds increased during the eight years of the study, and thus species communities changed towards warm-dwelling species (Table 1, Fig. 2). Model predictions for 500 m above sea level indicated a shift of communities towards average CTI values that are usually found at an altitude that is on average 8 m downhill from our study sites for plants (p = 0.010), 38 m downhill for butterflies (p = 0.006), and 42 m downhill for birds (p = 0.004; see Table 1). In other words, plant, butterfly and bird communities at 500 m apparently showed an average uphill shift of 8 m, 38 m and 42 m, respectively, within eight years. The change of plant communities at 500 m towards warm-dwelling species was thus 4.8 times slower compared to the change in butterflies (p = 0.021) and 5.3 times slower compared to the change in birds (p = 0.033). CTI changes of butterflies and birds were not significantly different (p = 0.415), with birds estimated to change 1.1 times faster than butterflies. The shifts in community averages of the three species groups at low altitudes were not accompanied by a decrease of community temperature variation CTV: while community variation in plants and birds apparently was largely stable over the study period, temperature niches of butterfly species in lowland communities even became more variable (Table 1, Fig. 2).

**Table 1:** Estimated parameter values from minimal adequate linear models on temporal changes in a) community average (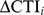) and b) community variation (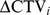) between 2003 and 2010 as a function of altitude, with linear (L), quadratic (Q) and cubic (C) polynomials of altitude as predictors and altitudinal ranges within 214 1-km^2^ sample squares as covariates.

	plants		butterflies		birds	
*a) community average* ΔCTI*_i_*						
Intercept	7.78	*	37.75	*	41.65	*
altitude (L)	−1.10×10^−2^	*	−4.59×10^−2^	*	4.09×10^−2^	
altitude (Q)	−3.00×10^−6^		−1.00×10^−6^		2.00×10^−6^	
altitude (C)	<1.00×10^−6^		<1.00×10^−6^		<1.00×10^−6^	
altitudinal range	−3.10×10^−2^		4.35×10^−2^		−2.00×10^−1^	
*b) community variation* ΔCTV*_i_*						
Intercept	−2.39		27.89	*	5.21	
altitude (L)	6.01×10^−2^		−1.10×10^−1^	*	3.91×10^−1^	
altitude (Q)	7.00×10^−5^		−1.50×10^−5^		2.80×10^−5^	
altitude (C)	<−1.00×10^−6^		<1.00×10^−6^		<1.00×10^−6^	
altitudinal range	−1.46×10^−1^		2.93×10^−1^	*	−3.39×10^−1^	

Intercepts indicate 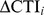 and 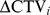 at 500 m above sea level. Asterisks (*) indicate significant values (p<0.05).

However, the rates of temporal changes towards warm-dwelling species decreased with altitude in plants and butterflies. At the highest altitudes, vascular plant and butterfly species communities even tended to change towards cold-dwelling species, although this trend was not statistically significant (Fig. 2): at 2350 m above sea level, which is the upper limit of the tree line in Switzerland [24], the models predicted a trend of plant and butterfly communities that was towards cold-dwelling species and towards CTI values that are usually found at an altitude that is on average 12 m uphill from our study sites for plants (p = 0.073), and 40 m uphill for butterflies (p = 0.055). In other words, plant and butterfly communities at the upper limit of the tree line showed a trend for an average downhill shift of 12 m and 40 m, respectively, within the eight years of the study.

In Fig. S1 we give the same results as in Fig. 2 but included all data points. Note that most apparent outliers in Fig. S1 were from sample squares with low species richness; because in the linear models, we accounted for the effect of species richness on heterogeneity of residuals, these data points had little influence on the results of the models. Accordingly, the effects of altitude on temporal change of plant and butterfly communities remained stable if outliers (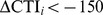 and 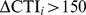 for plants; 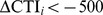 and 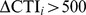 for butterflies) were excluded. In birds, the temporal change in CTI was not found to significantly depend on altitude, and the change of bird communities was towards warm-dwelling species at all altitudes (Fig. 2).

The increase in community variation that we found for butterflies in lowland communities decreased with altitude (Table 1, Fig. 2), and at higher altitudes, community variation for all three species groups did not significantly change over the study period (Fig. 2). Further, butterfly community variation showed a stronger temporal increase in sample squares with larger altitudinal ranges (Table 1). In all other analyses, altitudinal range within sample squares seemed not to affect the results, as in none of the statistical models altitudinal range had a significant effect on the temporal change of CTIs and CTVs (all p>0.108).

**Figure 3:**
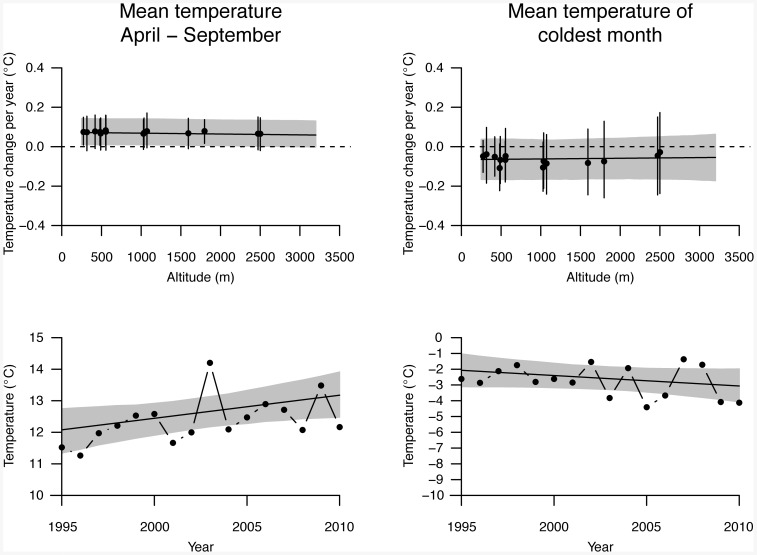
Temporal trends of air temperatures. Upper panels present temporal changes in mean temperatures for summer half-years (April to September, left panel) and for the coldest month (right panel) for the years 1995–2010 as depending on altitude. Points represent linear changes of temperatures over years, given in °C per year, for 14 meteorological stations distributed over Switzerland. Error bars are 95% confidence intervals, and grey areas represent 95% confidence intervals of the linear mixed model predictions for the average trend of temperature over years (solid line). Lower panels present mean temperatures for summer half-years (April to September, left panel) and for the coldest month (right panel). Solid lines indicate temporal trends as estimated from linear mixed models, and grey areas represent 95% confidence intervals of the model predictions.

The finding that temporal changes of plant and butterfly communities varied with altitude could not be explained by different temporal trends of air temperature at different altitudes: temporal trends in mean summer temperature and in mean temperature of the coldest month as measured at 14 meteorological stations were not found to vary with altitude (upper panels in Fig. 3; mean summer temperature: interaction temporal trend×altitude = −4.62×10^−6^, t = 1.51, p = 0.11; average temperature of coldest month: interaction temporal trend×altitude = −3.69×10^−6^, t = 0.23, p = 0.80). While summer temperatures increased over the years 1995–2010 by on average 0.07°C per year (Fig. 3; linear temporal trend = 0.07, t = 2.02, p = 0.048), mean temperatures of the coldest month did not significantly change over the years 1995–2010 (Fig. 3; linear temporal trend = −0.07, t = 1.24, p = 0.80).

## Discussion

In this study, we compared the temporal changes in average temperature indices of communities (CTIs) of vascular plants, butterflies and birds over an altitudinal range of about 2500 m. We found that in a rather short period of eight years (2003–2010), lowland communities of all three species groups changed towards warm-dwelling species. Such a change in communities was expected as resulting from current climatic warming.

In contrast, community temperature variation (CTV) was not found to decrease in any of the species groups, suggesting that climate warming has not led to more homogenous communities in terms of temperature niches of species. A trend towards more homogenous communities would be expected if due to climate warming, cold-dwelling species were decreasing without being replaced by warm-dwelling species, or if climate warming would promote a few ‘winning’ species at the expense of many other species [20], [35]. Rather, the change of lowland butterfly communities towards warm-dwelling species was accompanied by an increase in community variation. Variation in temperature niche breadths among species has been found to increase with increasing local variation of temperatures [36]; our study suggests that at least on the short term, variation in temperature niche breadths may also increase with warming air temperatures.

Although our data were collected on the same study sites during the same years for all three species groups, and thus species groups experienced largely the same overall environmental conditions, the community changes in CTI of butterflies and birds were about four to five times as fast as in plants. Still, the observed short-term shifts in plant communities seemed surprising, as short-term shifts of plant communities are mainly known from experimental studies that exposed plant communities to climatic conditions expected to occur at about the end of the century [37], [38]. Observational studies investigating entire communities in natural settings and suggesting shifts of plant communities over a period of just a few years seem to be scarce; so far, studies were mainly conducted at high elevations such as mountain summits [3], [4] or considered only selected plant species [13].

One main aim of our study was to investigate whether changes towards warm-dwelling species that are found in lowland communities (this study; [8], [9]) remain stable across the altitudinal gradient. We found that bird communities changed towards warm-dwelling species at similar rates at all altitudes. It seems likely that the uniform change of bird communities was due to warming air temperatures that were found over the entire altitudinal range (this study; [23]), rather than being mainly caused, e.g., by land-use changes that usually vary across the altitudinal range in Switzerland [39]. Strikingly, however, we found that temporal changes in CTI of plants and butterflies tended to decrease with altitude. Thus, while temporal changes of air temperatures were not found to depend on altitude, plant and butterfly communities changed towards warm-dwelling species at low altitudes but remained stable or even tended to change towards cold-dwelling species at high altitudes.

A decrease of changes in CTI with increasing altitude would be expected if the number of species with downhill range shifts were increasing with altitude. However, recent studies investigating species range shifts in relation to climate change mainly reported range shifts towards higher altitudes [3]–[6], [40]. Few studies also reported species moving towards lower altitudes [41], [42]. So far, most studies investigating altitudinal range shifts in alpine species focused on mountain summits. However, at mountain summits, it is by definition not possible to observe species from higher altitudes that have moved downhill, which could have led to a relative overestimation of species with uphill range shifts and underestimation of species with downhill range shifts. In contrast, our results are based on study sites that were randomly selected within the alpine environment, and based on those data, stable distributional ranges or even downhill range shifts at high altitudes seem to be more common than previously thought.

We can only speculate about potential mechanism that could have caused plant and butterfly communities to change towards warm-dwelling species at low altitudes but to remain relatively stable at higher altitudes. One reason could be that conditions in micro-habitats of alpine environments are often decoupled from conditions in the larger environment; this is due to the topographically induced variability of micro-climatic conditions that is usually much larger in alpine areas compared to lowland areas [16], [43], [44], and to the small size of alpine plants leading to communities that are aerodynamically decoupled from temperatures in the free atmosphere [43], [45]. Therefore, at higher altitudes, plant species do not necessarily need to shift their altitudinal ranges to escape climate warming [43], [46]. We thus predicted that temporal changes in CTIs of plants should be highest in the lowlands and should be decreasing with altitude, which was supported by our data. Butterfly communities showed a temporal change in CTIs that was similar to plants, probably because butterflies depend on their host plants for reproduction [47]. In contrast, birds with their larger body sizes and mobile behaviour are likely to be more strongly influenced by air temperatures than by micro-climatic conditions, and as a particularly mobile species group, birds have been shown to respond particularly fast to climate change [40]. This may explain why in contrast to plants and butterflies, bird communities changed towards warm-dwelling species across the entire altitudinal range of the study.

However, other mechanisms may equally likely explain our results. For example, possible downhill range shifts of alpine plant species may be explained by transient competitive release at the lower altitudinal margins of species distributions [41]. In alpine species, lower distributional margins are often characterised by intense competition among species [48], because species richness increases from alpine to subalpine areas [49]. Due to climate warming, degradation of permafrost at high altitudes increasingly leads to debris flow and landslides [41], [50]. Such habitat disturbances at lower distributional margins of alpine species might relax the role of competition as a selective filter for community assembly and could thus lead to downhill range shifts of alpine species [41] and therefore counteract the community effects of climate warming.

A further alternative cause for a temporal trend of plant and butterfly communities towards decreasing CTIs at higher altitudes could be land-use related habitat modification [51], [52]. For example, many pastures are now abandoned in the Swiss Alps, and trees are currently recolonizing subalpine grasslands [53]. It has been shown that abandonment of pastures could outweigh the effect of climate warming on species communities [53], [54]. If species temperature indices (STIs) of species that are promoted by land-use change are below the CTIs of communities that are present before a land-use change, this could lead to a decrease of CTIs over time that is not caused by climate change. Therefore, both climate warming and land-use change could generally be expected to affect CTIs, and they may do so in opposing directions [9], [55].

Manipulative experiments testing *a priori* hypotheses would be needed to make strong inference about mechanistic effects of global change and to disentangle effects of climate warming and land-use change on communities (for strong inference see [56]). Manipulative small-scale experiments, however, are hardly sufficient to draw conclusions on how multiple human pressures are affecting biodiversity in the real world; thus, understanding human impacts on natural biological systems requires surveys on biological change that is the integrated result of all human pressures over larger spatial scales [57], which is the focus of many long-term monitoring programs [58], [59]. Although biodiversity monitoring schemes usually have been implemented to assess spatial and temporal trends in biological systems without necessarily addressing underlying mechanisms [58], we here show that analysing data from such monitoring programs may at least help to develop hypotheses on mechanisms leading to biodiversity change [60].

Currently, most of the evidence for effects of climate warming on biodiversity comes from models forecasting future responses under different long-term scenarios for climate change [61], [62]. However, the temporal scales of such studies usually ranged from 20 to 100 years, considering biological consequences of climate change for periods of time that are far beyond the time frames in which policy makers are usually operating [63]. Here, we presented evidence that on the surprisingly short temporal scale of eight years, there were significant altitudinal shifts in communities of plants, birds and butterflies. We hope that our study contributes to fostering further research on short-term responses of local ecosystems to climate change that is urgently needed to set conservation practices [64]. Further, our results may support the idea that at least for plants and butterflies, alpine landscapes are buffering the effects of climate warming on species communities [43]. Whether such a buffering effect of alpine environments could be maintained over longer periods of time remains to be seen.

## Supporting Information

Text S1 **Field protocols for vascular plants, butterflies and breeding birds.**
(DOCX)

Figure S1 **Temporal change of temperature indices of plant, butterfly and bird communities.** The figure presents the same results as in Fig. 2, but additionally shows data points.
(DOCX)
