# Supplementary material for: Plants, Birds and Butterflies: Short-Term Responses of Species Communities to Climate Warming Vary by Taxon and with Altitude
Source: PLoS One. 2014 Jan 8;9(1):e82490. doi: 10.1371/journal.pone.0082490 (PMC3885385; doi:10.1371/journal.pone.0082490)
Supplement: Text S1 — Field protocols for vascular plants, butterflies and breeding birds. (DOCX) [file pone.0082490.s001.docx]

**S1.** **Field protocols for vascular plants, butterflies and breeding birds.**

Vascular plants

Plant surveys were performed by qualified botanists who received special training to reduce among-observer variation. For each sample square of 1 km^2^, occurrences (presence / absence) of vascular plant species were surveyed along a 2.5 km transect that followed existing trails wherever possible. If no trails existed, surveyors marked the transect route in the field and plotted it on a map. At a sample square in a given year, transects were inspected once in spring and again in late summer, assuring that data collection spanned a large variation in flowering phenologies that likely influenced species detection [[1](#_ENREF_1)]. Exceptions were sample squares at high altitudes with short vegetation period, where only one inspection per field season was conducted. During each inspection of a sample square, surveyors walked the transects in both directions and recorded all plant species within 2.5 m to each side of the transects on the way forth and back, respectively. Sampling characteristics of plant surveys were assessed in a previous study [[2](#_ENREF_2)]: two botanists independently assessed 23 transects; the mean of the relative differences between two assessments was 7.9%, corresponding to a difference of 19.7 ± 4.9 species (mean ± SE), with a total of 250 species recorded. For more information and quality measures on the field methods, see [[2](#_ENREF_2)] and [[3](#_ENREF_3)].

Butterflies

The field protocol for butterflies was based on the British butterfly monitoring scheme [[4](#_ENREF_4)]. Surveys of butterfly species occurrences (presence / absence) were performed by qualified entomologists who received special training to reduce among-observer variation. At a sample square in a given year, transects were inspected seven times between 21 April and 21 September in the lowlands, and four times between July and August above approximately 2000 m. The difference in numbers of inspections corresponds to the shorter flying season at higher altitudes; sites at high and low altitudes received approximately equal sampling effort per week of flight season, and differences in the number of visits should thus not bias the results [[1](#_ENREF_1)]. Surveys were conducted within separate time windows of 14 or 21 days, depending on a seasonal schedule, following a standardized protocol. Butterfly surveys were performed on the same transects as the surveys of vascular plants. Inspections of transects were conducted during favourable weather conditions, i.e. sunshine during more that 80% of the duration of the inspection, temperature of more than 13°C, and wind of less than 19 km/h (Beauford level 1-2). During each inspection of transects, surveyors walked the transects in both directions and recorded all day-flying butterfly species (including Hesperiidae and Zygaenidae) within 5 m to each side of the transects on the way forth and back, respectively. Detectability varied by species and averaged 88% per inspection [[5](#_ENREF_5)]. For more information and quality measures on the field methods, see [[5-8](#_ENREF_5)].

Breeding birds

Surveys and surveying methods were largely identical to the Swiss Ornithological Institute's common breeding bird survey [[9](#_ENREF_9),[10](#_ENREF_10)]. Bird surveys were performed by qualified volunteer ornithologists who used a territory mapping method [[11](#_ENREF_11)]. Breeding birds were surveyed using different transects than in vascular plants and butterflies, aiming at fully covering each sample square and at detecting all breeding bird species. This resulted in forested areas having greater transect lengths than did open sites. Transect lengths averaged 5.1 km (range 1.2 - 9.4 km). In inaccessible regions with very steep slopes, sample squares could sometimes not be fully covered. In such cases, any part of an area that was impossible to sample was marked on a map. Depending on altitude, each square was sampled two or three times between 15 April and 15 July. High altitude squares with less than 10% forest cover were only visited twice, corresponding to the shorter breeding season compared to low altitude squares. Mean detectability of birds across sites was estimated 89%, with a somewhat higher detectability at high altitude sites, in spite of receiving fewer visits [[10](#_ENREF_10)]. For more information and quality measures on the field methods, see [[9](#_ENREF_9),[10](#_ENREF_10),[12](#_ENREF_12)].

**References**

1. Pearman P, Weber D (2007) Common species detemine richness patterns in biodiversity indicator taxa. Biological Conservation 138: 109-119.

2. Plattner M, Weber D, Birrer S (2004) Data quality in monitoring plant species richness in Switzerland. Community Ecology 5: 135-143.

3. Wohlgemuth T, Nobis MP, Kienast F, Plattner M (2008) Modelling vascular plant diversity at the landscape scale using systematic samples. Journal of Biogeography 35: 1226-1240.

4. Pollard E, Moss D, Yates TJ (1995) Population trends of common british butterflies at monitored sites. Journal of Applied Ecology 32: 9-16.

5. Kéry M, Royle JA, Plattner M, Dorazio RM (2009) Species richness and occupancy estimation in communities subject to temporary emigration. Ecology 90: 1279-1290.

6. Kéry M, Plattner M (2007) Species richness estimation and determinants of species detectability in butterfly monitoring programmes. Ecological Entomology 32: 53-61.

7. Altermatt F, Birrer S, Plattner M, Ramseier P, Stalling T (2008) Erste Resultate zu den Tagfaltern im Biodiversitätsmonitoring Schweiz. Entomo Helvetica 1: 75-83.

8. Dorazio RM, Kéry M, Royle JA, Plattner M (2010) Models for inference in dynamic metacommunity systems. Ecology 91: 2466-2475.

9. Schmid H, Zbinden N, Keller V (2004) Überwachung der Bestandsentwicklung häufiger Brutvögel in der Schweiz. Sempach: Schweizerische Vogelwarte.

10. Kéry M, Schmid H (2006) Estimating species richness: calibrating a large avian monitoring programme. Journal of Applied Ecology 43: 101-110.

11. Bibby C-J, Burgess ND, Hill DA, Mustoe SH (2000) Bird census techniques. London: Academic Press.

12. Kéry M, Schmid H (2004) Monitoring programs need to take into account imperfect species detectability. Basic and Applied Ecology 5: 65-73.
