# Supplementary material for: Plants, Birds and Butterflies: Short-Term Responses of Species Communities to Climate Warming Vary by Taxon and with Altitude
Source: PLoS One. 2014 Jan 8;9(1):e82490. doi: 10.1371/journal.pone.0082490 (PMC3885385; doi:10.1371/journal.pone.0082490)
Supplement: Figure S1 — Temporal change of temperature indices of plant, butterfly and bird communities. The figure presents the same results as in Fig. 2, but additionally shows data points. (DOCX) [file pone.0082490.s002.docx]

**Figure S1. Temporal change of temperature indices** **of plant, butterfly and bird communities.** Given are model predictions for temporal changes of community average of temperature indices (${\Delta CTI}_{i}$, upper panels) and of community variation in temperature indices ($\Delta\text{CTV}_{i}$, lower panels) between two surveys at a sample square *i* separated by five years within the period 2003 - 2010, across the altitudinal range covered in the Swiss national biodiversity monitoring program. Points represent 214 1-km^2^ sample squares, and sizes of points are proportional to the number of species recorded during the first survey. Black lines are regression lines from minimal adequate linear models, and grey areas represent bootstrapped 95% confidence intervals. Predicted values with confidence intervals that do not include zero are judged as being significantly different from zero.
